# A Regulatory Perspective on a UK Federated Data Network for Medicines and Medical Devices: Lessons from a ‘Study-A-Thon’

**DOI:** 10.1007/s43441-025-00854-3

**Published:** 2025-08-19

**Authors:** Helen P. Booth, John Connelly, Daniel Dedman, Katherine Donegan, Alison Cave

**Affiliations:** Medicines and Healthcare products Regulatory Agency, 10 South Colonnade, Canary Wharf, London, E14 4PU UK

**Keywords:** Routinely collected health data, Government regulation, Data analytics, Medical device regulation, Real-world evidence, Real-world data

## Abstract

Internationally, medical regulators are seeking to make better use of real-world data (RWD) to support their decision making. While the UK National Health Service collects population-wide cradle-to-grave data, challenges remain around siloing, interoperability and access to data across different care settings. In 2023, a `Study-A-Thon’ was held to explore how mobilisation of a UK distributed data network might be used to generate real-world evidence (RWE) for regulatory purposes by increasing availability of RWD in a timely manner. Two research questions focusing on high-priority data gaps (medical devices and secondary care prescribing) were selected as case studies to support this work. This paper summarises details of the Study-A-Thon and discusses key learnings for the UK’s Medicines and Healthcare products Regulatory Agency (MHRA), UK stakeholders and international partners to reflect on when developing and implementing RWD strategies. Shortcomings of the data are discussed, such as a lack of follow-up for patients across care settings and the need to develop common data models to capture relevant information on medical product utilisation. The importance of local data and clinical expertise for success is highlighted, from encouraging better data collection at point of care through to appropriate interpretation of results. Successful delivery of results for both studies supports the view that, with further development, a UK federated data model could enhance national regulatory decision-making across the product lifecycle.

## Introduction

There is growing interest in use of real-world evidence (RWE) for improving public health in the field of medicines and medical devices regulation, with agencies developing systems to progress this agenda. RWE is derived from the investigation of real-world data (RWD) collected on patients through routine healthcare delivery. Studies conducted across multiple RWD sources offer the larger sample sizes needed to study rare exposures/outcomes and allow comparison across patient groups. However, these efforts have been hampered by barriers such as heterogeneity in the data collected, varying data quality, and different data governance requirements that result in inefficiency and long time-frames for study completion. Common data models (CDM) are being used to address some of these barriers by standardising the structure and terminologies used across datasets. Once standardised, shared quality assessments and analytical approaches can be deployed to generate RWE at pace and without the need to share patient-level data.

Internationally, the US Food and Drug Administration (FDA) Sentinel programme [1] has been supporting regulatory decision-making since 2016, while the European Union’s (EU) Data Analysis and Real World Interrogation Network (DARWIN EU) [2] completed development in 2023 and is now fully operational in its role supporting the evaluation work of the European Medicines Agency’s (EMA) scientific committees. Both schemes make use of centrally defined queries which are shared with locally distributed databases to answer safety-focused questions [3]. Both Sentinel and DARWIN EU utilise the Observational Medical Outcomes Partnership (OMOP) CDM, developed by the Observational Health Data Science and Informatics (OHDSI) network [3–5]. The OHDSI network promotes quality, transparency and efficiency in large-scale analytics for health data and has regional nodes worldwide, including the UK.

The UK is well-placed to initiate work in this area given the rich cradle-to-grave data it collects through its National Health Service (NHS), with nascent schemes underway [3, 4]. 

To explore the feasibility of generating RWE using a federated data network approach in the context of UK regulatory processes, the Medicines and Healthcare products Regulatory Agency (MHRA) held a regulatory Study-A-Thon in partnership with the OHDSI UK node and the University of Oxford. A Study-A-Thon is a ‘focused multiday research event’ [5] and this event aimed to explore data availability, timeliness and utility for a UK federated data network. Two study questions of high regulatory interest were investigated during the Study-A-Thon, on rectopexy using mesh devices for the treatment of rectal prolapse, and on prescribing of fluoroquinolones with a particular interest in secondary care. While the results of these studies have been published elsewhere, this paper reflects on learnings from the Study-A-Thon with regards to the use of UK RWE to support regulatory decision-making.

## Materials and Methods

The Study-A-Thon was held in Oxford over five days in 2023. The Pharmaco- and Device Epidemiology team at the Nuffield Department of Orthopaedics, Rheumatology and Musculoskeletal Sciences (NDORMS), University of Oxford, acted as coordinators of the event. They managed all logistics, invited collaborators to participate, and led on the analyses. A UK-based OHDSI node was established in 2023, and data partners were invited to join the Study-A-Thon from participating organisations.

Six data partners who had implemented conversion of their data into OMOP CDM format participated in the Study-A-Thon. A summary of the databases is given in Table 1, and included data from primary and secondary care, a specialist (paediatric) hospital and from regions across the UK.

**Table 1:** Data partners and databases included in the Study-A-Thon

Data partner	Type of data held & coverage period^1^	Number of subjects	Population
Barts Health	Secondary care inpatient, 2008–2022	2,643,160	Patients admitted to six hospitals in East London
CPRD^2^ Aurum	Primary care data linked to Hospital Episode Statistics, 1997–2021	44,851,398	Patients registered at participating GPs across England
CPRD^2^ GOLD	Primary care, 1987–2023	17,054,819	Patients registered at participating GPs across the UK
Great Ormond Street Hospital^3^	Secondary care, 2019–2022	135,511	Children treated at a paediatric hospital in central London
Health Informatics Centre^3^	Primary and secondary care (inpatient and outpatient), 1988–2022	1,190,126	Patients in Tayside and Fife
Lancashire Teaching Hospitals^3^	Secondary care inpatient, 2012–2022	1,793,793	Patients admitted to two hospitals in North-West England

^1^Coverage periods available at the time of the studyathon

^2^Clinical Practice Research Datalink (CPRD)

^3^Did not contribute data to the rectopexy study due to a lack of available data

The two study questions were selected after consultation with the MHRA. Topics were chosen on the basis of MHRA interest in evaluating a medical product’s benefit-risk profile and exploring novel data from a regulatory perspective. Study feasibility was also considered in terms of data availability and reuse of existing analytics. The studies were (a) a descriptive drug utilisation study of systemic fluoroquinolones, and (b) a characterisation of rectal prolapse and rectopexy (including use of mesh devices), including incidence and short-term outcomes. These questions offered the opportunity to investigate two priority data gaps: on medical devices and secondary care prescribing. The study objectives are described in Table 2. The study protocols were developed by colleagues at the MHRA and NDORMS, then adapted and submitted locally by each participating data partner according to their specific data governance procedures. The data analysis was conducted using R packages that were developed by NDORMS through DARWIN-EU^®^ funding. All code is available and using the following links: GitHub - https://github.com/oxford-pharmacoepi/FluroquinolonesTimeSeries; GitHub - https://github.com/LTHTR-DST/RectopexyStudyathon [6]. 

**Table 2:** Study-A-Thon questions and objectives

**Use of systemic fluoroquinolones in the UK: a drug utilisation study** Objectives: (a) To estimate trends in the incidence and prevalence of use of fluoroquinolones (overall and by ingredient) in the UK, stratified by calendar term/year and age for the period 2012–2022. The same analyses will be conducted using US data as a control. (b) To calculate the duration, indication and dose of fluoroquinolone use (at ingredient level) in the UK, stratified by calendar term/year, and age.
**Characterising rectal prolapse and rectopexy in the United Kingdom: population characteristics**,** incidence and surgical procedures** Objectives: (a) To describe the epidemiology of rectal prolapse over the study period (01/01/2013 to 31/03/2021), including (a) incidence and prevalence, and (b) baseline characteristics. (b) To describe the use of rectopexy over the study period, including trends over time (stratified by procedure type, age and sex) and baseline characteristics. (c) To estimate the risk of short-term (90 days) postoperative complications or further related procedures up to one-year post-index surgery.

The Study-A-Thon was attended in full by one to three representatives from each data partner and key personnel from the co-ordinating team NDORMS. Other NDORMS team members, representatives from the MHRA and clinicians from relevant specialities joined in part. The participants split into three groups, each led by an NDORMS team member, according to study question or to focus on analytics, with results reviewed throughout the week and refinements made based on any errors or suggested improvements identified. The results of the analyses were shared with participants on interactive web-based applications, with the aim of further development into manuscripts for publication and internal use by the MHRA and its expert committees.

## Results and Discussion

### Results

Results were produced for each study question and a brief summary of the high-level findings are presented in Table 3 as the results will be published separately. Two of the participating data partners, Great Ormond Street Hospital (GOSH) and Lancashire Teaching Hospitals, were using their OMOP-converted data to conduct research for the first time during the Study-A-Thon. Not all data partners were able to contribute to both study questions due to a lack of relevant data or ongoing conversion to OMOP (see Table 1).

**Table 3:** Headline findings from the Study-A-Thon for MHRA

**Use of systemic fluoroquinolones in the UK** • Prescribing trends produced for all six databases, including first description of secondary care prescribing and implementation of time series methods to explore impact of regulatory action in 2019. [7, 8] [6] • A reduction in primary care prescribing of fluoroquinolones was observed. In secondary care, prescribing of fluoroquinolones remained stable while use of other antibiotics increased. [4, 5]
**Characterising rectal prolapse and rectopexy in the UK** • Descriptive statistics produced on rectal prolapse and rectopexy (including short-term post-operative outcomes) for 3 of 6 databases. [9] [8] [6] • The incidence of rectopexy is decreasing, driven by a smaller proportion of patients with rectal prolapse being treated with surgery. • Complications post-surgery were high, with constipation the most common.

The pre-existing R packages used for the analyses were successfully applied to the UK OMOP format databases, but some modification of clinical phenotypes was required for appropriate selection of possible indications for antibiotic use and procedures/devices related to rectopexy. Small further adjustments were made to ensure that results could be released according to the data partner’s internal data governance rules.

The web-based applications used to share the study results give the viewer options to generate graphs and results tables with high granularity. Breakdowns are given by database, sex, age groups and clinical characteristics generating a large volume of results that can be challenging to navigate. When working with primary care data the denominator is the registered population. However, with secondary care data the population at risk is patients admitted to hospital, meaning that rates can be challenging to interpret and compare. The secondary care databases included in the Study-A-Thon incorporated dispensing data whilst the primary care data holds prescribing data. The dispensing data is more accurate in terms of understanding what has been administered but lacks details on dosage. The prescribing data does include dosage information, but misclassification can be introduced when the prescription is not collected or taken. Work was underway at some of the participating hospitals to map prescribing data. For the rectopexy study, Unique Device Identifiers (UDI) collected for administrative purposes were discovered in some of the source databases. These were incorporated into the Barts Health CDM during the Study-A-Thon, meaning information on the types of mesh inserted during rectopexy was available for the first time. It was not possible to utilise this data during the Study-A-Thon but this shows promise for future device research.

Findings from the rectopexy study were incorporated into a report presented to some of the MHRA’s expert advisory committees. The fluoroquinolones study provided valuable insight into the impact of risk minimisation measures (RMM) on prescribing in secondary care. With the lack of absolute reduction in fluoroquinolone use observed in secondary care, the Study-A-Thon results provided additional justification for the introduction of further measures in January 2024 [7, 10]. 

### Discussion: Regulatory Use Cases

The Study-A-Thon demonstrated that UK-databases in OMOP CDM format can be used to answer questions of regulatory interest. In this section, we describe regulatory use-cases where real-world evidence can be or is being used and discuss the value of a federated data network within these examples.

### Early Access to Medicines

A risk-proportionate and innovation-enabling approach to authorisation supports swift access to new medical products. To date, the greatest use of RWE in the pre-authorisation period has been via the use of external control arms in oncology trials and open-label extension studies [11], with use of RWD to facilitate trials of high interest to industry. Despite the absence of RWD on new products in the pre-authorisation period, direct regulatory needs can still be met by federated models through increased understanding of risk and disease epidemiology in target populations to contextualise experimental data. This requires detailed data on patient demographic and clinical characteristics. This information could be used to demonstrate disease severity and unmet need in applications to gain Promising Innovative Medicine (PIM) designation, and subsequently to investigate early adoption of the product in the Early Access to Medicines Scheme (EAMS) [12]. Where appropriate, MHRA is supportive of the use of RWE in the authorisation process, including as part of its innovative licencing schemes [13, 14]. RWE derived from federated analytics can underpin the development of feasible and proportionate risk management plans, as described by Gauffin et al., who used this approach to understand current treatment pathways, identify active comparators and estimate sample sizes for future pharmacoepidemiological studies [15]. Further, with improved secondary care data access expanded use of RWE in this space can contribute to a proactive vigilance strategy with rapid signal detection. Exploration of off-label prescribing for potential extensions to marketing authorisation may be feasible, as is development of external/historical control arms or in silico trial design.

### Signal Detection, Assessment and Contextualisation

Use of RWE as part of the signal detection and management process is not yet well-developed, with traditional processes relying on spontaneous reporting and expert assessment. A European Health Data and Evidence Network (EHDEN) Study-A-Thon demonstrated the feasibility of incorporating federated analyses of RWD into signal management procedures, but concluded further work was needed on the costs and benefits of integrating them into current practice [15]. Existing data governance rules can pose a barrier to RWD data access for signal detection purposes, due to requirements for well-defined protocols meaning analyses that are not targeted towards predetermined drug-event combinations are not accepted [8, 10, 15]. 

Advantages of increasing access to RWE for signal management include use of data with greater external validity than trial populations and the ability to target areas of missing information, such as patients with characteristics that preclude their participation in trials. Gauffin et al. demonstrated how federated analyses using OMOP-mapped data could provide context for signal assessors undertaking causality assessment [15]. Experience from the Study-A-Thon highlighted lack of linkage across care settings as a barrier to use of RWD for signal detection. For instance, signals with a product prescribed in primary care with adverse events presenting in secondary care (or vice versa) could not be identified in most of the available datasets. Use of a federated network does not overcome limitations of the underlying data that pose a barrier for signal assessment, including a paucity of long-term follow-up data and lack of an appropriate denominator in secondary care (based, as it is, on an inpatient population). Federated models can be leveraged effectively for signal contextualisation, including background incidence rates [16], drug and device utilisation and estimation of public health impact. Standardised methods could be developed for signal detection and prioritisation. Ensuring the included datasets are representative is key for this use.

### Benefit-Risk Evaluation

Use of RWE by regulatory agencies for the purpose of evaluating benefit-risk balance of medical products is well-established, with UK primary care datasets already highly utilised. Federated models allow regulators to harness heterogeneous data sources, including those covering specific sub-populations such as specialist hospitals like GOSH. From a UK perspective, a national data network would enable generation of local evidence at pace and permit focused analyses on patient groups stratified by characteristics associated with inequalities, such as geographic region or ethnicity. Early streamlining of analytics and governance processes is an important set-up element [17] and can support development of programmes for ongoing monitoring of products that could be outputted via dashboards. Issues with the available data remain, such as lack of linkage across settings and absence of long-term follow-up of patients treated in hospital. Both of these concerns were relevant to the rectopexy study, where non-acute outcomes from a secondary care-based procedure are likely to be reported in primary care, thus limiting the value of these datasets in isolation.

### Monitoring the Impact and Effectiveness of Regulatory Action

Understanding and communicating the effectiveness of RMMs will encourage continuous improvement in the application of regulatory action and build public trust. Time-series methods are of high value here [18], as demonstrated during the Study-A-Thon by the fluoroquinolones study. A federated data network can meet data needs for impact assessment, as data partners can be targeted to ensure priority patient groups are represented, plus variation in practice and the impact of RMMs between different care settings investigated. Better availability of secondary care data is needed, and development of the mapping process should be prioritised to ensure that hospital dosage data is present in the CDM to provide granular and accurate drug utilisation data. When interpreting results, the importance of understanding local context cannot be underestimated. For example, in the fluoroquinolones study, local antibiotic prescribing or formulary initiatives may have had equal or greater impact on observed rates than the RMMs circulated at a similar time that we would have been unaware of without the input of local experts. Buy-in from data partners is needed to facilitate the interpretation of results into accurate and actionable conclusions. Additionally, investment in ongoing engagement with data partners can facilitate improvements in the OMOP CDM and underlying data collection over time.

### Further Considerations

Having considered use cases where a federated model might offer value for UK regulatory purposes, there are a number of other considerations that deserve discussion.

As with all RWD, poor data quality can be a barrier to the generation of useful evidence. For regulatory purposes particularly important is lack of recording of non-serious adverse events and poor granularity of data. Improved recording of device utilisation is a high priority; key information, such as UDI’s, should be recorded at point of care and incorporated into the source electronic health record (EHR) for conversion to OMOP. A number of trusted device registries already exist, such as the National Joint Registry [19], and their use could be enhanced through linkage to EHR data. Ongoing initiatives such as Scan for Safety and development of NHS England’s Outcomes and Registries Programme (ORP) may support development in this area [20, 21]. 

Setting up a federated data network demands careful selection of data partners. From a regulatory perspective, data should be representative of the population according to key demographic variables such as age, sex, ethnicity and socioeconomic status, and across health care settings to allow a complete overview of the services where medicines and medical devices are deployed. From a more practical perspective, the data partners need to be engaged with the process and ideally enable local clinician involvement to support the timely interpretation of study findings. This highlights the need for financial resource and sustainability when considering set-up of a network. For the proof-of-concept Study-A-Thon, a small number of partners with mapped data were included, but to demonstrate real value it will be necessary to achieve greater population coverage.

Data partners who participated in the Study-A-Thon had taken different approaches to conversion of source data into OMOP CDM. Voss et al. highlighted the need for a multidisciplinary team with expertise in the source data, OMOP and its vocabularies and technical skills to accomplish this successfully [17]. Standardisation of these approaches among the community will be important in ensuring reliability of the data, and minimising time and resource needed for conversion. Developing the right mix of data science and subject matter expertise in this area should be a priority. Federated analyses produce huge volumes of results, so careful consideration is needed on the format for outputting relevant and digestible results, thereby facilitating interpretation. Finally, the patient voice must be heard in the design of studies and interpretation of their results, so resource should be dedicated to applying best practice in this area [22]. 

## Conclusions

The MHRA’s Data Strategy lays out our commitment to using data to deliver proportionate and robust regulatory decisions in a timely fashion, with evaluation of the role of CDMs and federated analyses being a part of this ambition [9, 11, 23]. The proof-of-concept regulatory Study-A-Thon demonstrated the possible value of a federated data model for regulatory purposes in the UK by completion of two studies within a short timescale, both of which offered novel insights into key data gaps (secondary care prescribing and device data). The importance of working with local data experts and clinicians throughout the study was a key learning, as it ensured results were interpreted correctly and could be turned into actionable insights. Engagement with those working at point of care will also support capture of relevant and high-quality data at source. Further, continual updates to the OMOP CDM should be leveraged to better capture relevant information on devices, such as UDI. The growing engagement with OHDSI and OMOP in the UK will support this work, with the NHS Secure Data Environment adopting OMOP as its CDM of choice. Increasing expertise in data science and experience of dataset conversion will support consistent and thorough data mapping, as well as opportunities to harness new datasets.

To successfully leverage federated analyses to support regulatory decision-making careful thought must be given to the curation of datasets, with representative and high-quality data required to offer un-biased evidence on the benefits and risks of medical products. Seizing new opportunities for patient-level linkage and integration of data across healthcare settings to ensure the necessary capture of exposures and clinically relevant outcomes will be important. Recommendations from the Sudlow Review around increasing availability of NHS and non-health sector data, and on a UK-wide approach to data access and governance, will be beneficial to this agenda [24]. Also relevant are recommendations made in Expert Reports for the UK COVID-19 Inquiry around improved data access, linkage and infrastructure for efficient RWE generation in a future pandemic [25, 26]. International efforts to utilise federated analytics for regulatory purposes are now coming to fruition, and the Study-A-Thon demonstrated the possibilities for a similar approach in the UK. This approach will be developed further by HDRUK’s pilot Real-World Evidence Network [3, 10, 27], and opportunities for international collaboration will be possible. With further development and resources, a UK federated data network has the potential to bolster regulatory decision-making that is both fast-paced and scientifically robust, thus enhancing our ability to protect patients and improve public health.

## Data Availability

No datasets were generated or analysed during the current study.
